# MYC/BCL2/BCL6 triple hit and TP53 deletion in a case of high-grade B cell lymphoma receiving CAR T cell immunotherapy

**DOI:** 10.1136/jitc-2020-002029

**Published:** 2021-06-01

**Authors:** Jiachen Wang, Zhen Shang, Jue Wang, Jinhuan Xu, Weigang Li, Yuqi Guan, Li Yang, Wei Zhang, Kefeng Shen, Meilan Zhang, Jin Wang, Liting Chen, Qinlu Li, Cheng He, Na Wang, Liang Huang, Yi Xiao, Min Xiao, Jianfeng Zhou

**Affiliations:** 1 Department of Hematology, Tongji Hospital of Tongji Medical College of Huazhong University of Science and Technology, Wuhan, Hubei, China; 2 Department of Orthopedics, Tongji Hospital of Tongji Medical College of Huazhong University of Science and Technology, Wuhan, Hubei, China

**Keywords:** immunotherapy, adoptive, hematologic neoplasms, case reports

## Background

Immunotherapy has become one of the most effective treatments for refractory/relapsed (r/r) B cell lymphoma.^1^ In particular, chimeric antigen receptor (CAR) T cell immunotherapy has recently been found to be a highly effective treatment for r/r diffuse large B cell lymphoma (DLBCL).^2^

High-grade B cell lymphoma (HGBL) with translocations of MYC, BCL2, or/and BCL6 detected by FISH or standard cytogenetics, which is defined by the 2016 WHO Classification of Tumors of Hematopoietic and Lymphoid Tissue as Double- and Triple-Hit HGBL (HGBL-DH/TH),^3^ remains a treatment challenge. HGBL makes up approximately 8% of DLBCL, among which 16% is HGBL-TH,^4^ and abnormal expression of *TP53* is one of the risk factors for HGBL. Several studies have reported poor outcomes of these aggressive lymphomas treated with first-line and second-line chemotherapy. Moreover, the National Comprehensive Cancer Network (NCCN) guidelines have recommended clinical trials such as CAR T cell immunotherapy,^5^ and several anti-CD19 CAR-T trials have been reported. In the phase I JULIET trial, the response rate of double-hit lymphoma patients was 50%, and the complete response rate was 25% at 3 months,^6^ while in the ZUMA-1 trial, the best CR rate for double-hit lymphoma patients was 64% at 12 months.^7^ The prognosis of HGBL-DH/TH patients was poorer than that of normal DLBCL patients. Previously, we reported the safety and efficacy of CD19/22 CAR T cell cocktail immunotherapy alone^8–11^ and following ASCT^12^ in our center. This therapeutic regimen can improve the long-term outcome of r/r double hit lymphoma.

There is no consensus in the rule of CAR T cell therapy for HGBL-TH patients thus far. Here, we present the first MYC/BCL2/BCL6 triple-hit lymphoma patient receiving murine and human CAR T cell therapy and expect to provide insights into the therapeutic strategy for such patients.

## Case report

A 38-year-old man presented to a local hospital in September 2018 with intermittent abdominal pain for 1 month. He was diagnosed with ⅣA stage (Ann Arbor staging system) primary abdominal DLBCL. Immunohistochemistry (IHC) indicated that BCL2, BCL6, CD19, CD20, CD10, C-MYC, and TP53 were positive in his initial diagnosed lymph node, whereas CD3, CD5, and CD30 were negative, consistent with germinal center B origin. He received a standard dose of R-CHOP (rituximab, cyclophosphamide, vincristine, adriamycin, and prednisone) for six cycles but experienced progressive disease. The patient then received intensified induction chemotherapy of one cycle of R-ESHAP (rituximab, etoposide, cytarabine, cisplatin, and methylprednisolone), but the disease progressed again.

**Figure 1:**
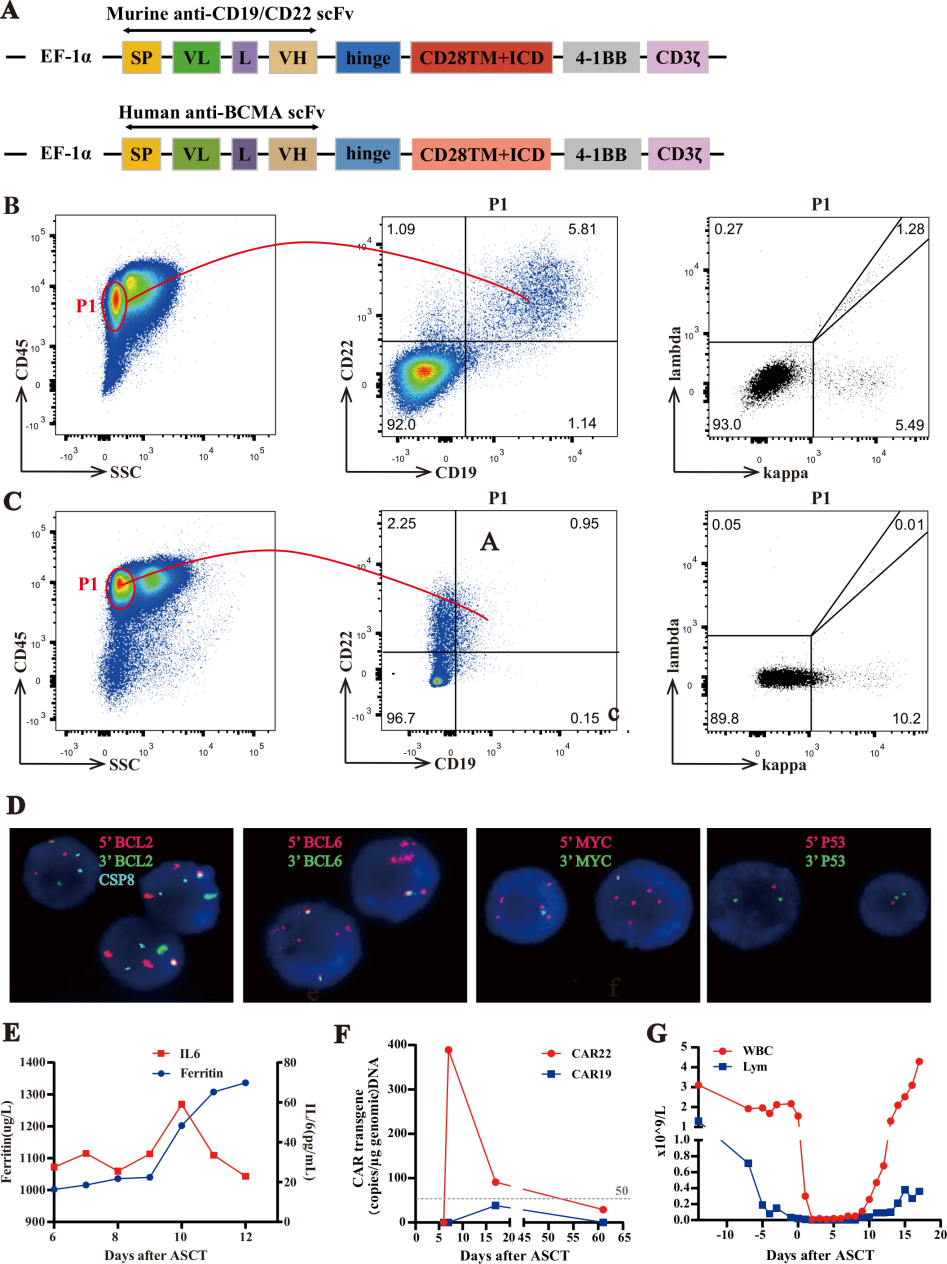
Clinical examinations in the sequential infusion of murine anti-CD19 and anti-CD22 CAR T cell therapy. (A) Schematic diagram of murine CAR-19/CAR-22 vectors and human CAR-BCMA vectors. (B–C) Phenotypic analysis of ascites before murine CD19 and CD22 CAR T infusion. The P1 gate represented live lymphocytes. In the P1 gate flow cytometry confirmed CD19+ and CD22+ in figure 1F and CD19− and CD22+ in figure 1G, with kappa restriction. (D) Fluorescence in situ hybridization of ascites (BCL2/CSP18 (18q21) probe, BCL6 (3q27) break apart probe, C-MYC (8q24) break apart probe, P53/CSP17 (17p13), 1000×). (E) Levels of IL-6 and ferritin after CAR T cell therapy. (F) CAR-19 and CAR-22 transgene copy numbers detected by ddPCR. (G) Dynamic WCC numbers and lymphocyte numbers before and after CAR T cell therapy. ASCT, autologous hematopoietic stem cell transplantation; CAR, chimeric antigen receptor; IL, interleukin; SP, single peptide; VH, variable H chain; VL, variable L chain; WCC, white cell count.

The patient was referred to our hospital in March 2019 to receive ASCT and CAR T cell therapy. Before treatment, target antigen expression for CAR T therapy (CD19, CD20, CD22, and BCMA) was confirmed in initially diagnosed lymph nodes by immunohistochemical staining (online supplemental figure 1A). He was enrolled in a clinical trial of sequential cocktail infusion of murine anti-CD19 and anti-CD22 CAR T cells following ASCT. The patient was given a standard dose of the BEAM regimen (300 mg/m^2^ bis-carmustine, −6 days; 200 mg/m^2^ etoposide, −5 to −2 days; 400 mg/m^2^ cytarabine, −5 to −2 days; and 140 mg/m^2^ melphalan, −1 day) as myeloablative chemotherapy.^13^ CAR19 and CAR22 T cells were infused 6~7 days (May 8, 2019–May 9, 2019) after autologous stem cell infusion (day 0, May 2, 2019). The structure (figure 1A), manufacture, and preparation of CAR T cells and hematopoietic stem cells were described as previously.^10^ The patient had stable disease on day +58 (June 27, 2019), as evaluated by enhanced abdominal CT compared with that on the day before stem cell collection. PET/CT scan on day +71 (July 12, 2019) indicated that mediastinum and abdominal lymphomas had progressed, and new lesions had appeared in the groin. Subsequently, the patient received sintilimab injection^14^ (an anti-PD-1 antibody) plus other immunotherapeutic drugs (rituximab, chidamide, bortezomib, and dexamethasone) from day +78 (July 19, 2019) to day +99 (August 9, 2019). At the same time, the disease continued to progress rapidly. After ASCT and CAR19/22 T cell cocktail therapy, the CD19 antigen was lost (figure 1B, C). Nevertheless, IHC indicated positive expression of BCMA in tumor cells. The patient was then transferred to another hospital to receive an infusion of human-derived anti-BCMA CAR T cells for compassionate use. Unfortunately, the disease remained refractory. The timeline of the main events in the clinical treatment of this patient is presented in online supplemental table 1.

10.1136/jitc-2020-002029.supp1 Supplementary data

10.1136/jitc-2020-002029.supp2 Supplementary data

Before CAR T cell treatment, various analyses were performed to study the molecular features of the patient. Rearrangements of the monoclonal receptor genes IGKA and IGKB were detected in the bone marrow (online supplemental figure 1B). Next-generation sequencing (NGS) was performed on his initial diagnosed formalin-fixed paraffin-embedded inguinal lymph node biopsy. One germline mutation (*PIM1*, c.403G>A, p.Glu135Lys, 49.40%) and three somatic mutations (*TP53* missense mutation, c.706T>G, p.Tyr236Asp,80.6%; *KMT2D* frameshift mutation, c.15547del, p.Val5184Cysfs*59, 47.0%; *IGLL5* missense mutation, c.64G>A, p.Glu22Lys, 47.58%) were detected. Germline DNA was extracted from leukocytes. Sanger sequencing confirmed that the patient carried the PIM1 mutation in a heterozygous state (online supplemental figure 1C). After CAR T cells infusion, large amounts of tumor-derived ascites appeared, and FISH analysis found MYC, BCL2, and BCL6 (triple-hit) rearrangements with *TP53* gene deletion (figure 1D) on day +81 (July 22, 2019). In addition, karyotypic analysis suggested a complex karyotype as follows: 92~93, XXYY, t(3;14)(q27;q32)×2,6, der(8)t(2;8)(p12;q24)×2,11, add(11)(q23), del(17)(p13) ×2, der(18)t(14;18)(q32;q21)×2,+2mar[cp10] (online supplemental figure 1E).

After sequential infusion of anti-CD19 and anti-CD22 CAR T cells, grade 1 cytokine release syndrome (CRS) was observed, and interleukin-6 and ferritin increased slightly and transiently (figure 1E). Neither the anti-CD19 nor the anti-CD22 CAR transgene expanded or persisted in this case(figure 1F), compared with the previously reported average level in our center,^8^ and dropped quickly to baseline on day +17 (May 19, 2019). With CAR T cells disappearing quickly, white cell counts and lymphocytes returned to their previous levels on day +17 (figure 1G). Flow cytometry analysis of ascites showed that CD19 and CD22 markers were positively expressed in lymphoma cells on day +78 (July 19, 2019), but CD19 antigen was lost by 1 month later, on day +110 (August 20, 2019) (figure 1B, C).

**Figure 2:**
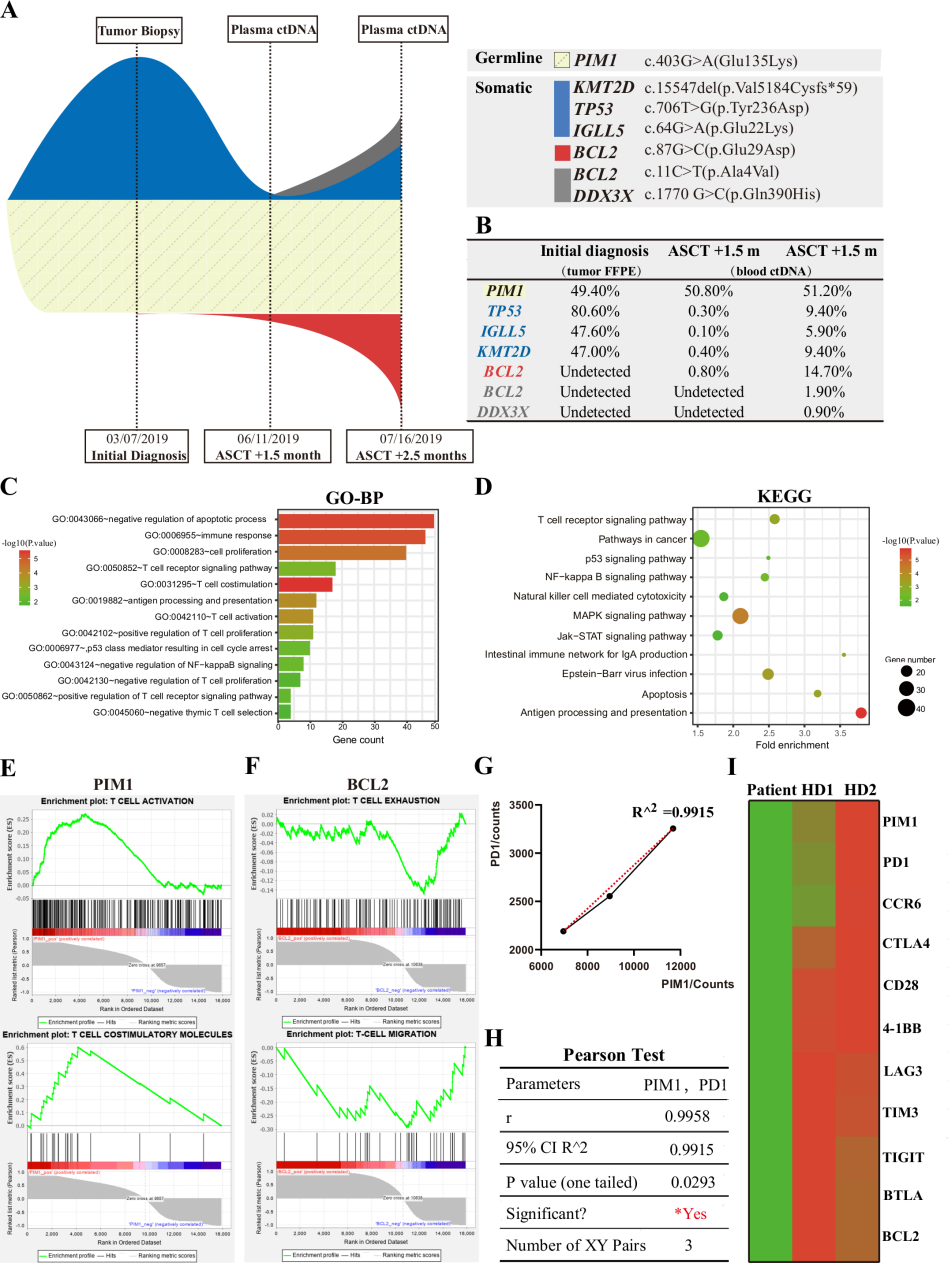
DNA and RNA high-throughput sequencing results and analysis. (A) Schematic models of evolutionary progression before and after CAR T cell infusion. Germline clones, primary dominant clones, secondary dominant clones, and subclones are represented in creamy white, blue, red, and gray shapes, respectively. (B) References and VAFs of DNA mutations investigated by NGS. An NGS panel with 157 target genes was employed for analysis of the FFPE sample. The mean amplicon coverage of the patient’s FFPE sample was 11,204.9×, and its uniformity of coverage (Pct >0.2*mean) was 95.29%. A panel with 171 target genes was employed for analysis of peripheral blood ctDNA samples. ctDNA NGS was performed by duplex unique molecular identifier sequencing technology. The mean coverage of the target region are 151,775.17× and 116,533.09×. The mean coverage of DS211 on the target region was 1911.26× and 1734.61×. (C) The histograms show the top 13 GO-BP enrichment results of differentially expressed genes between the patient and two healthy donors. The x-axis represents the enriched gene count, and the intensities of different colors represent the p value. (D) The bubble diagram shows the top 11 KEGG enrichment items of differentially expressed genes between the patient and two healthy donors. The x-axis represents the gene ratio, and the intensities of different colors represent the p value. (E) GSEA of the PIM1 gene. GSEA for T cell activation sets and T cell costimulatory molecule sets based on Pearson correlation with PIM1 expression level. Bottom: the plot of all genes. Y-axis, the value of the ranking metric; x-axis, the RANK for all genes. (F) GSEA of the BCL2 gene. GSEA for T cell exhaustion sets and T cell migration sets based on Pearson correlation with *BCL2* expression level. Bottom: the plot of all genes. Y-axis, the value of the ranking metric; x-axis, the rank for all genes. (G) The correlation plot showed a strong correlation between the expression of PIM1 and PD1 (R^2^=0.9915). (H) Positive correlation between the expression of PIM1 and PD1 (r=0.9958, p<0.05, Pearson test). (I) Heat map of RNA expression on T cell activation-related and exhaustion-related genes. Columns represent the patient and two healthy donors. FFPE, formalin-fixed paraffin-embedded; GSEA, gene set enrichment analysis; NGS, next-generation sequencing; VAFs, variant allele frequencies.

To explore the mechanisms underlying this persistent refractoriness, we performed additional genetic and transcriptional studies retrospectively. In addition to NGS performed on tumor biopsy at the initial diagnosis, peripheral blood circulating tumor DNA (ctDNA) NGS was performed twice and revealed potential clonal evolution (figure 2A, B). When the patient’s abdominal pain had been relieved after CAR T cell therapy, on day +40 (June 11, 2019), ctDNA NGS indicated a secondary primary clone of *BCL2* mutation (c.87G>C, p.Glu29Asp). One month later, when lymphoma had progressed and abdominal pain had reappeared, on day +75 (July 16, 2019), a primary tumor-derived clone with *TP53* missense mutation, *KMT2D* truncating mutation, and *IGLL5* missense mutation reamplified rapidly, and a new subclone with *BCL2* and *DDX3X* mutations emerged.

In contrast, patient T cells sorted by CD3+ magnetic beads (Miltenyi, CD3+ MicroBeads) from peripheral blood mononuclear during hematopoietic stem cell collection before the ASCT were used for transcriptional analysis. The GO and KEGG analysis of differentially expressed genes between the patient and two healthy donors indicated enrichment in T cell costimulation and activation pathways and in JAK/STAT, NF-KB and MAPK signaling pathways (figure 2C, D). To explore the immune responses in which key genes were involved, an immune gene set for CAR T cell characterization (nCounter CAR-T Characterization Panel, NanoString) was identified by gene set enrichment analysis (GSEA), and Pearson’s correlation coefficient was used as a metric. GSEA (Pearson) indicated that genes whose expression was positively correlated with PIM1 were enriched in T cell activation and costimulatory molecules (figure 2E), and those positively correlated with BCL2 were enriched in T cell exhaustion and migration (figure 2F). Moreover, the expression of the *PIM1* gene was significantly correlated with that of the T cell exhaustion gene PD1 (figure 2G, H). In addition, the expression of immune-related proteins in the patient’s T cells was relatively lower than that in healthy controls (figure 2I).

## Discussion

HGBL-DH/TH is a phenotypically and genetically heterogeneous disease with a dismal prognosis. Several studies have recently revealed MYC/BCL2/BCL6 rearrangements and TP53 mutations as independent indicators of prognosis in patients with DLBCL treated with standard chemotherapy. And TP53 mutation predispose to a poor outcome in children with B-ALL treated with hCD19 CAR T cells.^15^ The NCCN guidelines have recommended clinical trials such as CAR T cell immunotherapy. However, the mechanism underlying its resistance to various therapies remains inconclusive.

First, tumor antigen escape is often seen in failure for long-term disease control in CAR T cell therapy. In this case, although CD19 antigen expression was lost after treatment, infusions of CD19-targeted and CD22-targeted CAR T cell products cocktails might partially overcome this phenomenon. Interestingly, clonal evolution with the appearance of new *BCL2* mutation and subclones with *DDX3X* and *BCL2* mutations was detected in the plasma ctDNA after CAR T cell therapy. The *BCL2* mutation in the secondary clones might be related to T cell dysfunction (figure 2E, F). Furthermore, It is highly suspected that this case was a genetic composite, with features of EZB-MYC^+^ and A53 according to the 2020 LymphGen algorithm.^16^ This composite genetic subtype exhibits relatively lower immune signatures and is associated with poorer outcomes, as confirmed by RNA-seq (figure 2H). In conclusion, the pathogenic molecular background, antigen escape, and clonal evolution may contribute to resistance to CAR T cell therapy in this case. Moreover, it is worth noting that plasma ctDNA NGS is useful as a noninvasive method to monitor the clonal evolution process.

Second, intrinsic T cell function is vital for patient response to CAR T therapy. Specific characteristics of CAR T cell subtypes in the infusion product are correlated with better therapeutic results.^17^ Enhanced transcription of genes associated with T cell activation and memory T cells may predict a better response to T cell expansion and persistence. Furthermore, Lynn *et al*^18^ found that a functional deficiency in c-Jun mediates dysfunction in exhausted human T cells which indicates that deficiency of crucial genes may extensively influence T cell function. Patients with no response (NR) have a poor response in terms of the persistence and expansion of cellular kinetics. PIM1 plays an essential role in regulating signal transduction cascades, which promote cell survival, proliferation, and drug resistance. In addition, *PIM1* somatic mutation was identiﬁed as one of the potential early drivers of lymphomagenesis.^19^*PIM1* (c.403G>A, p.Glu135Lys, legacy Identifier in COSMIC: COSM1161628) is frequently reported in DLBCL in somatic states, and this mutation was predicted to be damage by FATHMM and SIFT. However, in this case, the patient carried the *PIM1* germline mutation in the heterozygous state, whether the *PIM1* germline mutation was associated with the lack of amplification or persistence of CAR T cells, and the abnormal transcriptional features of the patient’s CD3^+^ T cells in terms of T cell antigen presentation, activation, and costimulation pathways has yet to be determined. Unfortunately, this patient passed away and could only be studied retrospectively from RNA sequencing. For patients with immunodeficiency-related mutations, allogeneic HSCT can be considered a better option than autologous HSCT, which can compensate for the defects of intrinsic lymphocyte immunodeficiency. According to NCCN Guidelines V.1.2021 for acute myeloid leukemia (age ≥18 years), patients with a family history of leukemia, hematologic cancer or abnormalities together with the presence of genetic mutations should be considered for germline testing or genetic counseling. It is strongly recommended that patients with a germline variant allele frequency of 40%–60% be referred for germline testing. However, germline variants in CAR T cell immunotherapy have been poorly studied; further research is needed to investigate the impact and may be applied for guiding clinical therapy and donor selection.

In conclusion, several studies have recently revealed MYC/BCL2/BCL6 rearrangements and TP53 mutations as independent indicators of prognosis in patients with DLBCL treated with standard chemotherapy; EZB-MYC^+^ and A53 subtypes are associated with poorer prognosis; and the germline mutation of PIM1 (c.403G>A, p.Glu135Lys) is reported for the first time and is highly suspected that this germline mutation was related to T cell defect. The factors previously make this an intractable case and may contribute to CD19 antigen loss and T cell defect. Tumor-derived antigen escape and T cell dysfunction, two interactive factors, are major challenges to CAR T cell therapy. Tumor burden and antigen density can positively stimulate T cell expansion, which in turn increases the risk and severity of CRS. Such a dilemma should be carefully considered for each patient. We propose that precise monitoring of tumor genetic abnormalities and T cell function should be conducted during CAR T cell therapy. Antigen loss, clonal evolution, and T cell defects might appear simultaneously and contribute to resistance in combination.

## References

[1] Cheson BD, Pfistner B, Juweid ME, et al. Revised response criteria for malignant lymphoma. J Clin Oncol 2007;25:579–86. 10.1200/JCO.2006.09.2403 17242396

[2] Lin JK, Muffly LS, Spinner MA, et al. Cost effectiveness of chimeric antigen receptor T-cell therapy in multiply relapsed or refractory adult large B-cell lymphoma. J Clin Oncol 2019;37:2105–19. 10.1200/JCO.18.02079 31157579

[3] Swerdlow SH, Campo E, Pileri SA, et al. The 2016 revision of the world Health organization classification of lymphoid neoplasms. Blood 2016;127:2375–90. 10.1182/blood-2016-01-643569 26980727 PMC4874220

[4] Aukema SM, Siebert R, Schuuring E, et al. Double-hit B-cell lymphomas. Blood 2011;117:2319–31. 10.1182/blood-2010-09-297879 21119107

[5] Zelenetz AD, Gordon LI, Abramson JS, et al. NCCN guidelines insights: B-cell lymphomas, version 3.2019. J Natl Compr Canc Netw 2019;17:650–61. 10.6004/jnccn.2019.0029 31200358

[6] Schuster SJ, Bishop MR, Tam CS, et al. Tisagenlecleucel in adult relapsed or refractory diffuse large B-cell lymphoma. N Engl J Med 2019;380:45–56. 10.1056/NEJMoa1804980 30501490

[7] Nastoupil LJ, Jain MD, Feng L, et al. Standard-Of-Care Axicabtagene Ciloleucel for relapsed or refractory large B-cell lymphoma: results from the US lymphoma CAR T Consortium. JCO 2020;38:JCO1902104:3119–28. 10.1200/JCO.19.02104 PMC7499611 32401634

[8] Wang N, Hu X, Cao W, et al. Efficacy and safety of CAR19/22 T-cell cocktail therapy in patients with refractory/relapsed B-cell malignancies. Blood 2020;135:17–27. 10.1182/blood.2019000017 31697824

[9] Cao W, Wei J, Wang N, et al. Entecavir prophylaxis for hepatitis B virus reactivation in patients with CAR T-cell therapy. Blood 2020;136:516–9. 10.1182/blood.2020004907 32291456

[10] Wei J, Zhu X, Mao X, et al. Severe early hepatitis B reactivation in a patient receiving anti-CD19 and anti-CD22 CAR T cells for the treatment of diffuse large B-cell lymphoma. J Immunother Cancer 2019;7:315. 10.1186/s40425-019-0790-y 31753002 PMC6868854

[11] Chen L, Xu B, Long X, et al. Car T-cell therapy for a relapsed/refractory acute B-cell lymphoblastic lymphoma patient in the context of Li-Fraumeni syndrome. J Immunother Cancer 2020;8:e000364. 10.1136/jitc-2019-000364 32345625 PMC7213909

[12] Wei J, Mao Z, Wang N, et al. Long-Term outcomes of relapsed/refractory double-hit lymphoma (r/r DHL) treated with CD19/22 CAR T-cell cocktail therapy. Clin Transl Med 2020;10:e176. 10.1002/ctm2.176 32997409 PMC7507504

[13] Cuccuini W, Briere J, Mounier N, et al. MYC+ diffuse large B-cell lymphoma is not salvaged by classical R-ICE or R-DHAP followed by beam plus autologous stem cell transplantation. Blood 2012;119:4619–24. 10.1182/blood-2012-01-406033 22408263 PMC3815438

[14] Hoy SM. Sintilimab: first global approval. Drugs 2019;79:341–6. 10.1007/s40265-019-1066-z 30742278

[15] Pan J, Tan Y, Deng B, et al. Frequent occurrence of CD19-negative relapse after CD19 CAR T and consolidation therapy in 14 TP53-mutated r/r B-ALL children. Leukemia 2020;34:3382–7. 10.1038/s41375-020-0831-z 32346068

[16] Wright GW, Huang DW, Phelan JD, et al. A probabilistic classification tool for genetic subtypes of diffuse large B cell lymphoma with therapeutic implications. Cancer Cell 2020;37:e14:551–68. 10.1016/j.ccell.2020.03.015 PMC8459709 32289277

[17] Deng Q, Han G, Puebla-Osorio N, et al. Characteristics of anti-CD19 CAR T cell infusion products associated with efficacy and toxicity in patients with large B cell lymphomas. Nat Med 2020;26:1878–87. 10.1038/s41591-020-1061-7 33020644 PMC8446909

[18] Lynn RC, Weber EW, Sotillo E, et al. C-Jun overexpression in car T cells induces exhaustion resistance. Nature 2019;576:293–300. 10.1038/s41586-019-1805-z 31802004 PMC6944329

[19] Juskevicius D, Lorber T, Gsponer J, et al. Distinct genetic evolution patterns of relapsing diffuse large B-cell lymphoma revealed by genome-wide copy number aberration and targeted sequencing analysis. Leukemia 2016;30:2385–95. 10.1038/leu.2016.135 27198204

