# Correction: MYC/BCL2/BCL6 triple hit and TP53 deletion in a case of high-grade B cell lymphoma receiving CAR T cell immunotherapy

**DOI:** 10.1136/jitc-2020-002029corr1

**Published:** 2022-03-11

Wang J, Shang Z, Wang J, *et al*. MYC/BCL2/BCL6 triple hit and TP53 deletion in a case of high-grade B cell lymphoma receiving CAR T cell immunotherapy. *J Immunother Cancer* 2021;9:e002029. doi:10.1136/jitc-2020-002029

In the original published article, the funding statement was incomplete. The “(No. 8170211 to MX)” lost a number.

The correct funding statement is: This work was supported by the National Natural Science Foundation of China (No. 81 770 211 to MX) and the National Natural Science Foundation of China (No.81830008 to JZ).

