# Supplementary figures and images for: MYC/BCL2/BCL6 triple hit and TP53 deletion in a case of high-grade B cell lymphoma receiving CAR T cell immunotherapy
Source: J Immunother Cancer. 2021 Jun 1;9(6):e002029. doi: 10.1136/jitc-2020-002029 (PMC8173290; doi:10.1136/jitc-2020-002029)

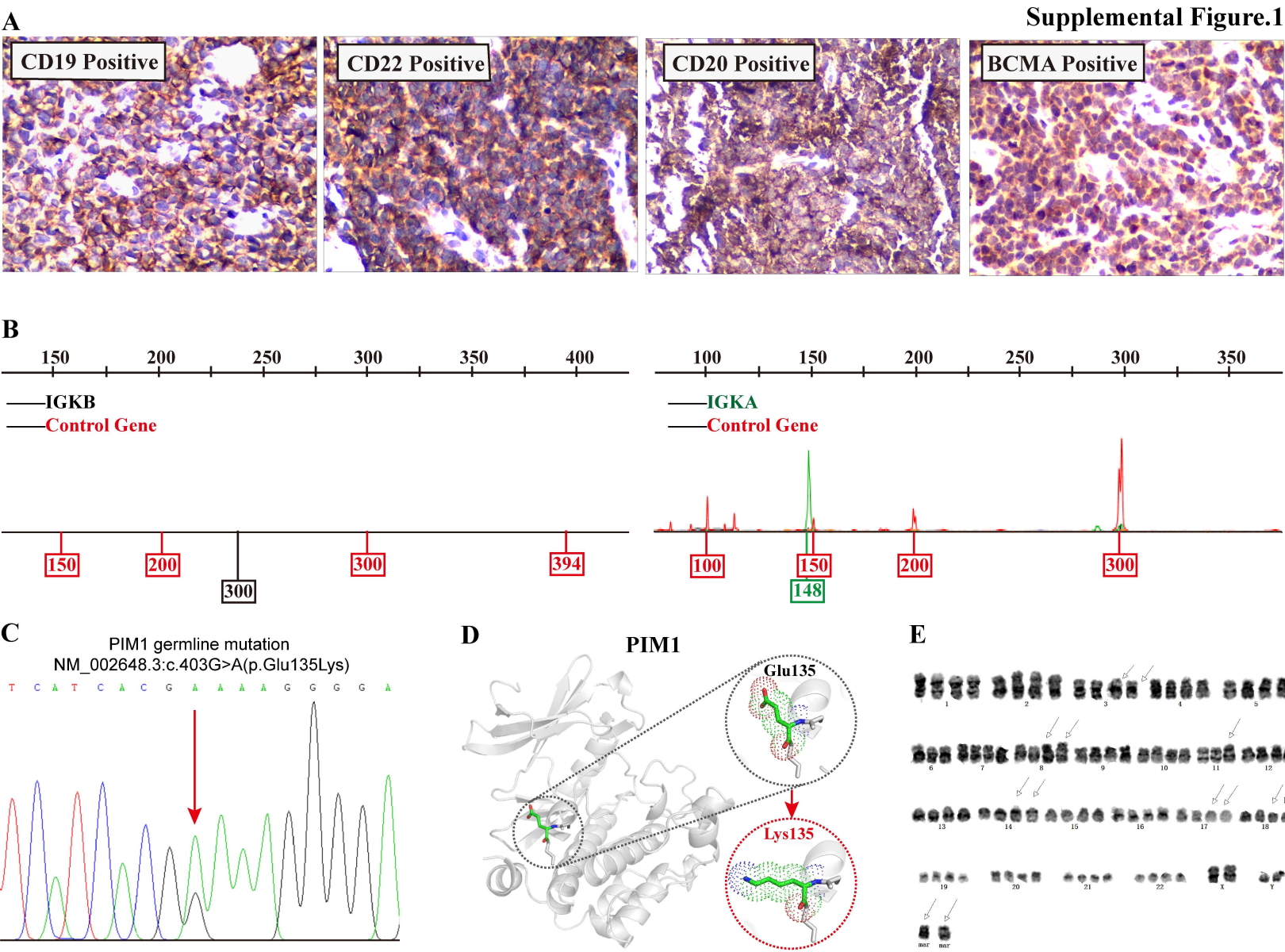

Supplement: Supplementary data [file jitc-2020-002029supp001.pdf]
