# Supplementary material for: MYC/BCL2/BCL6 triple hit and TP53 deletion in a case of high-grade B cell lymphoma receiving CAR T cell immunotherapy
Source: J Immunother Cancer. 2021 Jun 1;9(6):e002029. doi: 10.1136/jitc-2020-002029 (PMC8173290; doi:10.1136/jitc-2020-002029)
Supplement: Supplementary data [file jitc-2020-002029supp002.pdf]

Supplemental Table 1 Brief chronology of key clinical events in this case

Time before and after CAR T cell therapy	Date	Key events
-8.5 months	09/14/2018	Diagnosis of DLBCL, GCB
-8 months to -3 months	09/19/2018 to 01/22/2019	6 cycles of ***R-CHOP*** (response evaluation: PD)
-2.5 months	02/19/2019	1 cycle of ***R-ESHAP*** (response evaluation: PD)
-3 weeks	03/15/2019	1 cycle of ***ICE***
-9 days to -1 day	04/25/2019 to 05/01/2019	Lymphocytes collection and myeloablative chemotherapy regimen (***BEAM***)
0 day	05/02/2019	***ASCT***
+6 day	05/08/2019	***Murine anti - CD22 CAR T cell therapy*** (2.77 x 10^6 cells/kg)
+7 day	05/09/2019	***Murine anti - CD19 CAR T cell therapy*** (2.50 x 10^6 cells/kg)
+1.5 month	06/27/2019	Response evaluation by CT : SD
+2 months	07/12/2019	Response evaluation by PET/CT : PD
+2.5 months to +3 months	07/19/2019 to 08/11/2019	***Sintilimab Injection*** (an anti PD-1 antibody) plus novel immunotherapeutic drugs (***rituximab, chidamide, bortezomib and dexamethasone***)
+5 months	10/02/2019 to 10/06/2019	Lymphodepleting chemotherapy regimen (***FC***) and ***human anti-BCMA CAR T cell therapy***
+6 months	11/05/2019	Response evaluation by PET/CT : PD
